# Supplementary material for: Metabolic Footprinting of Microbial Systems Based on Comprehensive In Silico Predictions of MS/MS Relevant Data
Source: Metabolites. 2022 Mar 17;12(3):257. doi: 10.3390/metabo12030257 (PMC8949988; doi:10.3390/metabo12030257)
Supplement: Supplementary file 1 [file metabolites-12-00257-s001.zip › ESM_1.pdf]

ESM1

Additional plots

Metabolites

Metabolic footprinting of microbial systems based on comprehensive in-silico predictions of MS/MS relevant data

Alexander Reiter[1,2], Jian Asgari[1,2] , Wolfgang Wiechert[1,3], Marco Oldiges[1,2]

[1] Forschungszentrum Jülich GmbH, Institute of Bio- and Geosciences, IBG-1: Biotechnology, Jülich 52425, Germany

[2] RWTH Aachen University, Institute of Biotechnology, Aachen 52062, Germany

[3] RWTH Aachen University, Computational Systems Biotechnology, Aachen 52062, Germany

Corresponding author: Prof. Dr. Marco Oldiges, mail: m.oldiges@fz-juelich.de, phone: +49 2461 61-3951, fax: +49 2461 61-3870

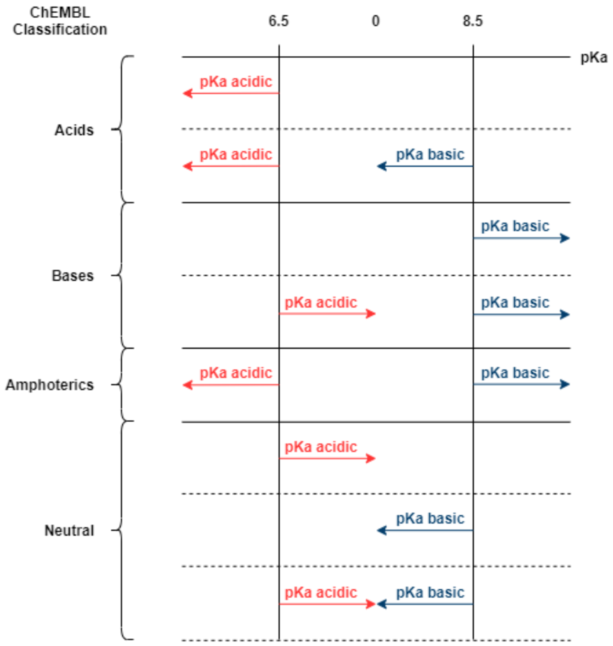

Chart 1: Visual representation of ChEMBL pK_a classification; Classification of acids, bases, amphoterics (zwitterions) and neutral molecules based on their acidic and basic pKa values.

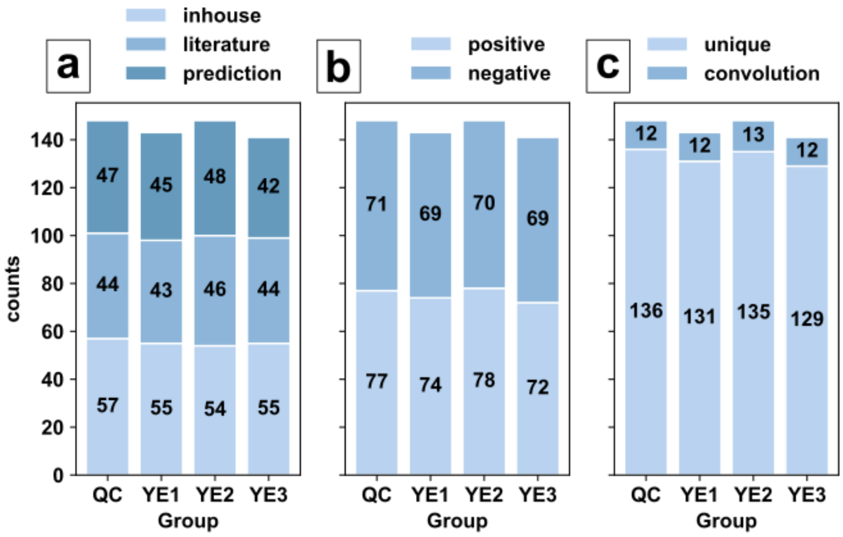

Chart 2: Metabolite identification and classification of yeast extract case study for quality control samples (QC) and three yeast extracts (YE1, YE2, YE3); Classification based on (a) Source of mass transition, (b) ionization mode, (c) Unique and convoluted mass transitions.

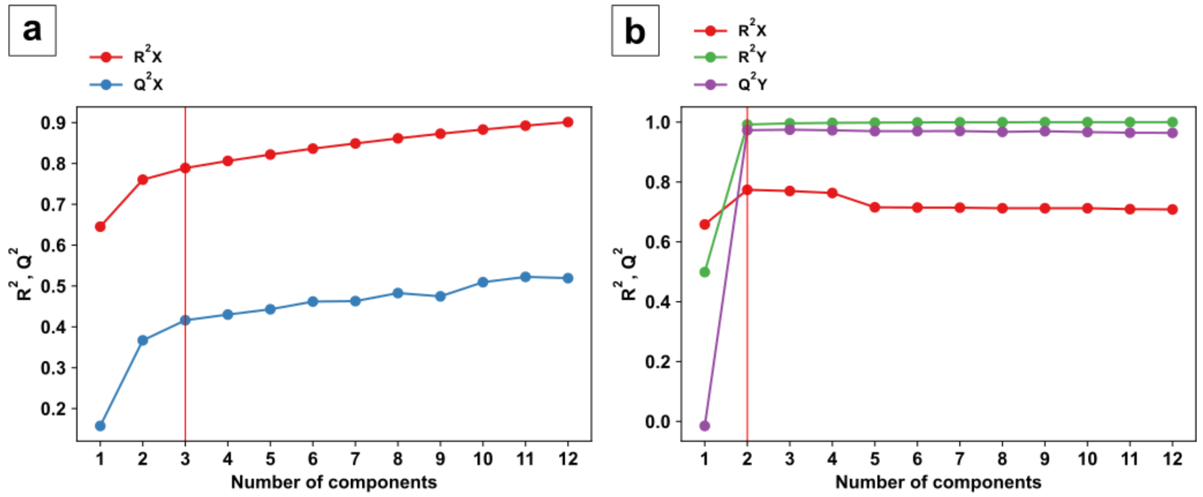

Chart 3: Scree plots for hyperparameter optimization of PCA (a) and PLS-DA (b); Model performance indicator were determined by stratified double 5-fold cross-validation. R2X describes the goodness of fit or explained variance of the predictor matrix by the model. Q2X describes the goodness of prediction. R2Y describes the goodness of fit or explained variance of the response by the model. Q2Y describes the goodness of prediction for the response. The hyperparameter optimization was stopped, if the increase in Q2X (a) or Q2Y (b) was less than 5%

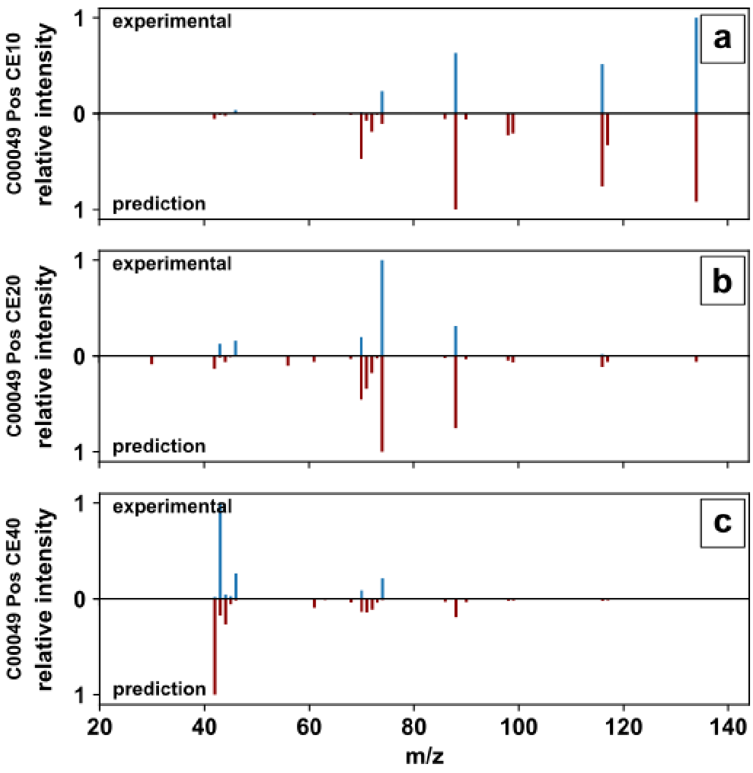

Chart 4: Mass-to-charge spectra comparison for C00049 in positive mode; Experimental data acquired by direct infusion of metabolite standard with ESI-QqToF in product ion scan mode for 10 (a), 20 (b) and 40 V (c). Predicted product ion spectra acquired by CFM-ID based on SMILES compound identifier

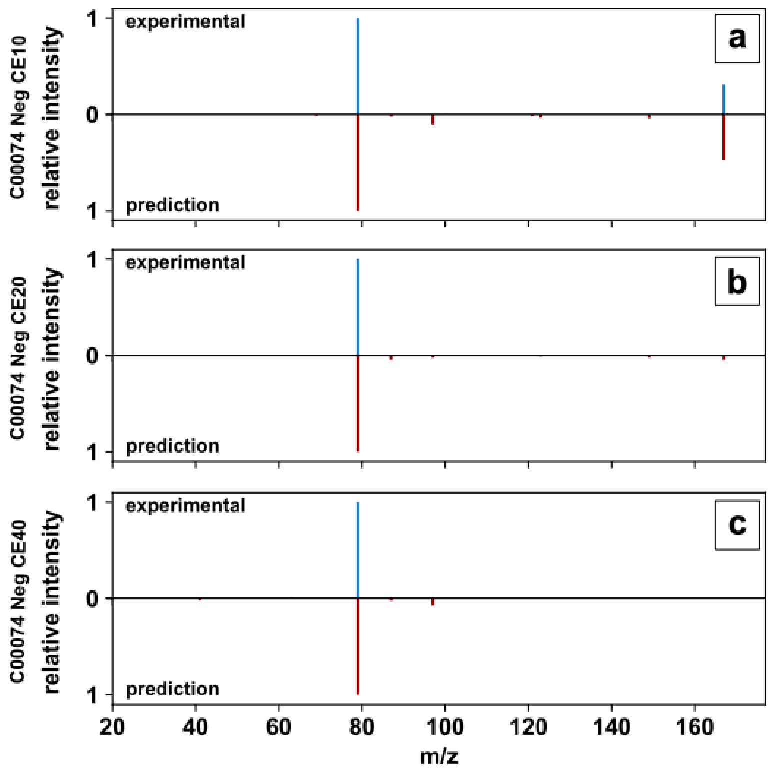

Chart 5: Mass-to-charge spectra comparison for C00074 in negative mode; Experimental data acquired by direct infusion of metabolite standard with ESI-QqToF in product ion scan mode for 10 (a), 20 (b) and 40 V (c). Predicted product ion spectra acquired by CFM-ID based on SMILES compound identifier

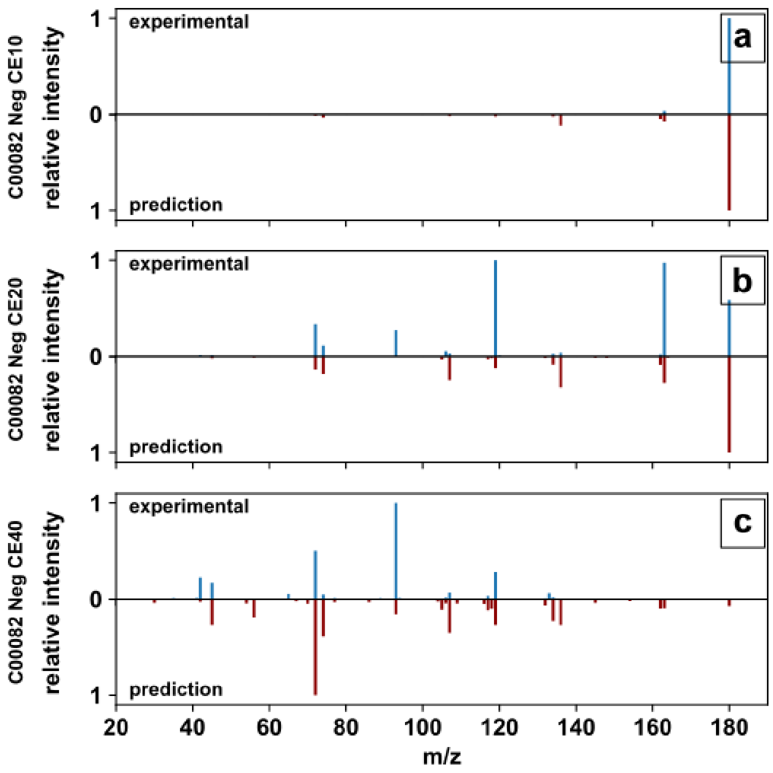

Chart 6: Mass-to-charge spectra comparison for C00082 in negative mode; Experimental data acquired by direct infusion of metabolite standard with ESI-QqToF in product ion scan mode for 10 (a), 20 (b) and 40 V (c). Predicted product ion spectra acquired by CFM-ID based on SMILES compound identifier

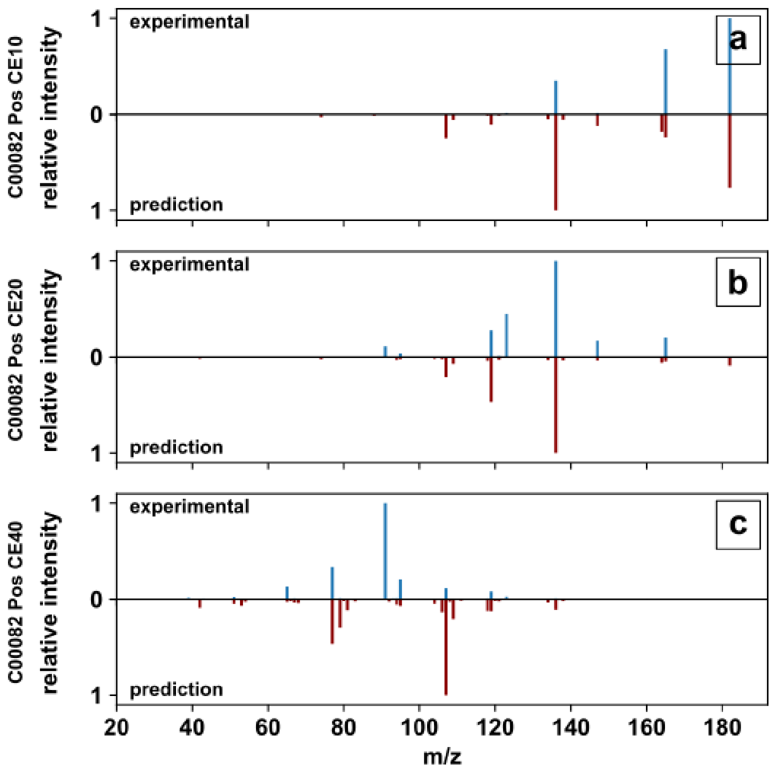

Chart 7: Mass-to-charge spectra comparison for C00082 in positive mode; Experimental data acquired by direct infusion of metabolite standard with ESI-QqToF in product ion scan mode for 10 (a), 20 (b) and 40 V (c). Predicted product ion spectra acquired by CFM-ID based on SMILES compound identifier

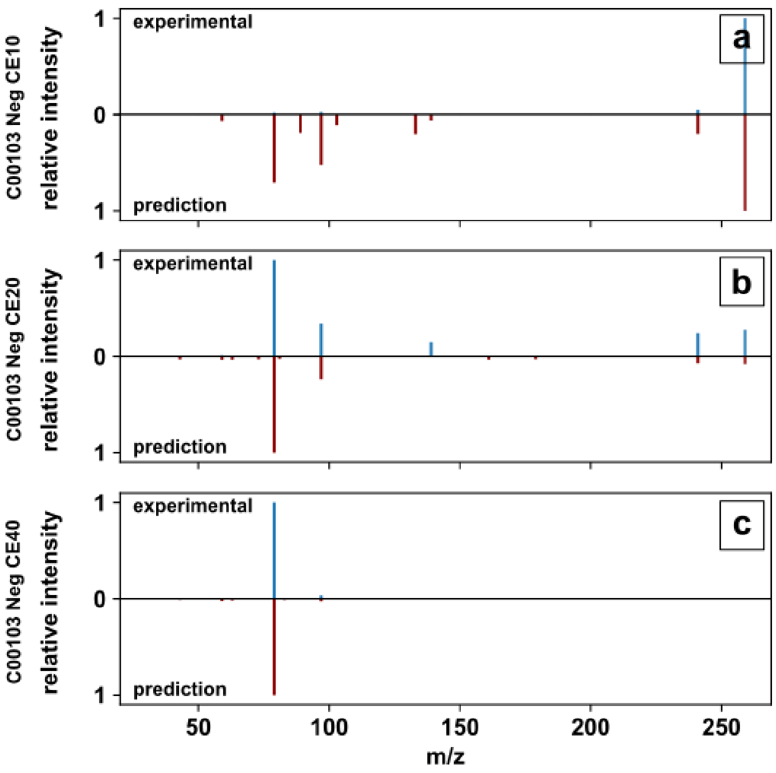

Chart 8: Mass-to-charge spectra comparison for C00103 in negative mode; Experimental data acquired by direct infusion of metabolite standard with ESI-QqToF in product ion scan mode for 10 (a), 20 (b) and 40 V (c). Predicted product ion spectra acquired by CFM-ID based on SMILES compound identifier
